# Consumer access to health information on the internet: health policy implications

**DOI:** 10.1186/1743-8462-2-13

**Published:** 2005-06-28

**Authors:** W Guy Scott, Helen M Scott, Terry S Auld

**Affiliations:** 1 Department of Applied And International Economics, Massey University at Wellington, Private Box 756, Wellington, New Zealand; 2 ScottEconomics, PO Box 14403, Kilbirnie, Wellington, New Zealand

## Abstract

**Background:**

Providers of health care usually have much better information about health and health care interventions than do consumers. The internet is an important and rapidly evolving source of global health-related information and could provide a means of correcting for asymmetric information. However, little is known about who accesses this information and how it is used in New Zealand.

The aims of this research were to: determine the nature of the health information sought, how respondents use the information, how helpful they perceive the information to be, and the self-assessed value of such information.

**Methods:**

The researchers conducted an anonymous five minute telephone and mall intercept survey of randomly selected Wellington residents who had searched for health-related information on the internet. Investigators entered the data into an Excel spreadsheet and transferred it to SPSS for data cleaning, data exploration and statistical analysis. Search time costs were based on the opportunity cost of income foregone and respondents were asked to provide a money value for the information found.

**Results:**

Eighty-three percent of respondents accessed the internet from home, and 87% conducted the search for themselves. Forty-five percent of people were looking for general health and nutrition information, 42% for data about a specific illness and 40% for a medicine.

After finding the information, 58% discussed it with a family member/ friend/ workmate, 36% consulted a general practitioner, 33% changed their eating or drinking habits, and 13% did nothing. Respondents found the information very quick to find and useful. It took them on average 0.47 hours and cost $12 (opportunity cost of time) to find the information. The average value of the data found was $60 and the net benefit to the consumer was $48 ($60 – $12).

**Conclusion:**

The results of this research could assist providers of health information via the internet to tailor their websites to better suit users' needs. Given the high perceived value of internet health information (greater than the average general practitioner fee) and the fact that some of the information found may be unreliable or even unsafe a valuable public health policy initiative would be to provide an improved New Zealand health information website containing information on how to evaluate data sourced from the world-wide-web and links to a range of useful and trustworthy health information sites.

## Background

### Asymmetric information

Asymmetric information is the term used to describe a situation where different decision makers have different information. Providers of health care usually have much better information about health and health care interventions than do consumers. Health care is frequently provided under asymmetric information conditions [1-3] and providers may capture the market and disadvantage consumers. Asymmetric information is a serious cause of market failure and has attracted the attention of some of the world's leading economists [4].

Uncontrolled and unregulated free markets may fail to yield the optimal outcome for society (with respect to society's goal of allocative efficiency). If markets are not efficient and if markets do not exist for all goods, the "invisible hand" [5] of the market place will not maximise social welfare. Welfare is maximised only when marginal social benefit and marginal social cost are equated in all markets.

**Figure 1:**
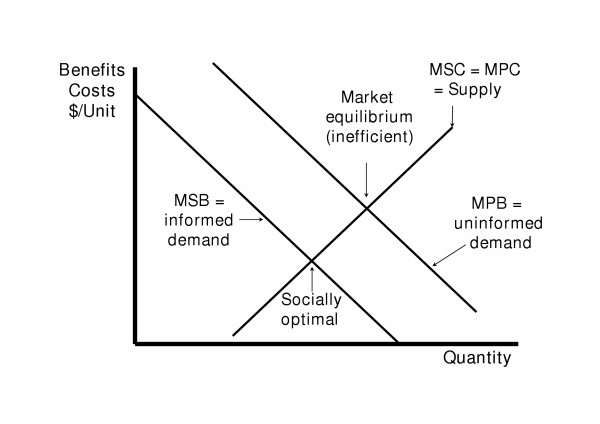
**Asymmetric information and market failure. **MSB = marginal social benefit, MPB = marginal private benefit, MSC = marginal social cost, MPC = marginal private cost. In this example, the uninformed market demand curve lies to the right of and above the informed demand curve. Market equilibrium is inefficient and results in consumers paying a higher price and consuming more than if they were fully informed.

If health care providers have more and better quality information than consumers, and if the health service/product (medical consultation, medicines, surgery, medical products) appears to the consumer to be of higher quality and more appropriate than it actually is then consumers will demand more than they would if they had better information. In other words, the "uninformed" demand curve lies above and to the right of the "informed" demand curve (see figure 1). The end result is that consumers will pay a higher price and consume more of the service than with the perfect information situation (which would be allocatively efficient if all other conditions of perfect competition were met). Situations could also occur where the "uninformed" demand curve is below and to the left of the "informed" demand curve.

Correcting for market failure caused by imperfect information is a common reason for government intervention in markets. However, frequently the costs of government regulation and control outweigh the benefits and there is a danger that in attempting to fix one problem others may be created or magnified [6]. In addition, some government intervention may be justified but too much is inefficient (as government intervention increases the marginal costs of intervention will eventually become greater than the marginal benefits).

### The internet

The internet is an important and rapidly evolving source of global health-related information. Health information is one of the most commonly searched topics on the internet [7] but little is known about who accesses this information in New Zealand and how it is used.

New Zealand has a high proportion of internet users. At the end of 2004, 60% of the population were internet users [8,9]. Fifty-three percent of households in Wellington City have access to the internet compared with 34% for New Zealand as a whole [10]. International comparisons in 2003 show New Zealand had 5263 internet users per 10,000 population compared with 5667 in Australia, 5514 in the US, 5128 in Canada and 4231 in UK [11].

If the quality of information provided on the internet could be evaluated and if consumers found such information useful the internet could provide a partial low cost solution to the problems of asymmetric information. Accordingly, the aims of this research were to; determine the nature of the health information sought, how respondents use the information, how helpful they perceive the information to be, and the self-assessed value of such information.

## Methods

A pilot questionnaire was developed, tested and refined using a small number of respondents. Quantitative data was collected using a mix of telephone and face-to-face mall intercept interviews [12]. Mall intercept interviews were conducted in the central city business district and in a range of large suburban shopping centres. Two data collection modalities were adopted to reduce bias and widen the target audience. Both methods enabled the interviewer to discuss and elaborate on the questions and to probe for clear answers. The survey was conducted in Wellington, New Zealand during November and December 2004. Selection criteria for the survey were that respondents must be over the age of 15 years and have searched the internet for health-related information.

The questionnaire asked who would use the information, the type of data sought, actions taken as a result of the search, health professionals recommending an internet search, search time, usefulness, value of the information, search location and demographics (gender, age and income). Age and gender breakdowns for the Wellington region were obtained from Statistics New Zealand [13]. Income classifications were the current New Zealand personal income tax brackets.

Usefulness of information found was assessed on a five point rating scale (1 no use at all, 2 somewhat useful, 3 useful, 4 very useful, and 5 extremely useful) and although reference or tie points were described, respondents were not restricted to whole numbers. The perceived benefits of the information found were quantified by asking respondents to provide a money value for the data uncovered. Search costs were assessed from the income of each respondent and search time (cost = search time multiplied by annual income divided by 47 working weeks divided by 40 hours per week). Costs concerned with ownership of computing equipment and access to the internet were regarded as sunk and thus not relevant to the incremental cost of obtaining information. It was assumed that respondents would have 47 working weeks (New Zealand employment legislation provides for 3 weeks annual leave and 2 weeks statutory holidays) and would work 40 hours per working week. Income for the group under $38,000 was assumed to be $18,999 (mid point of range), income group $38,000 to $60,000 was $49,000 (mid point of band), and income for group over $60,000 was assigned $80,000.

After editing and coding, data were entered into an Excel [14] spreadsheet and transferred to SPSS 12.0.1 for Windows [15] for detailed statistical analysis. Descriptive statistics were calculated for all variables and where appropriate, confidence intervals were estimated and F, t, and Chi-square tests applied to the data.

Sensitivity analysis was conducted by altering the magnitude of the perceived value, annual income and search time, and recording the effect on net benefits. This was necessary because some respondents may not have been able to accurately estimate search time and value, and income estimates were subject to error as income data was collected in the form of taxation income brackets rather than specific values.

## Results

Usable questionnaires were obtained from 126 respondents. Almost three quarters (71%) of respondents were aged between 26 to 65 years and over half (56%) of the sample were females. Thirty-six percent had incomes less than $38,000 per annum, 37% between $38,000 and $60,000 and 26% had incomes greater than $60,000. Table 1 presents the demographic profile of the sample. The majority of respondents (83%) searched the internet from home, 33% at work and 8% at other locations (table 2). Most people (87%) were searching for themselves, 49% for a family member, and 4% were looking on behalf of friends, neighbours, or co-workers (table 3). Responses may add to more than 100% because some respondents may have given more than one answer.

**Table 1:** Sample demographics of those who had searched the internet for health information

Demographic	Wellington		Sample
	%	N	%
Age			
16 to 25 years	17.2%	20	15.9%
26 to 45 years	39.4%	55	43.7%
46 to 65 years	29.7%	34	27.0%
Over 65 years	13.8%	17	13.5%

Total	100.0%	126	100.0%

Chi-square value 1.03, probability that the population and sample are the same 0.79.			

			
Gender			
Female	52.1%	71	56.3%
Male	47.9%	55	43.7%

Total	100.0%	126	100.0%

Chi-square value 0.9, probability that the population and sample are the same 0.34.			

			
Income			

Under $38,000		36	36.4%
$38,000 to $60,000		37	37.4%
Over $60,000		26	26.3%

Total		99	100.0%

**Table 2:** Search location

	**N**	**%**
Home	105	83.3%
Work	42	33.3%
Educational institution	5	4.0%
Library	4	3.2%
Neighbour	1	0.8%

Total	126	124.6%

Responses add to more than 100% because respondents gave more than one answer.

**Table 3:** Person for whom search undertaken

	N	%
Self	109	86.5%
Family member	62	49.2%
Friend/ neighbour/ workmate	5	4.0%

Total	126	139.7%

Responses add to more than 100% because some respondents gave more than one answer.

The three most common reasons for searching the internet were for general health or nutrition information (45% of respondents), a specific illness (42%), and a medicine (40%). On average, searchers were looking for two topics. The mean search time was 0.46 hours, the mean value placed on the information was found to be $61, and usefulness score 3.2 (on a five point rating scale). When comparisons were made across types of information sought, little difference in value or usefulness was found. There was a statistically significant (p < 0.05, t test) difference between the time taken to find information about a specific illness (0.52 hours) and that for sports and fitness topics (0.34 hours) (table 4).

**Table 4:** Type of health information sought by search time, value and usefulness

**Information sought**	**Number seeking**		**Search time**		**Value**	**Usefulness**
	**N**	**%**	**Hours**		**$**	**Score (a)**
General health and nutrition	57	45.2%	0.45		65	3.19
A specific illness	53	42.1%	0.52	(b)	65	3.19
Medicine	51	40.5%	0.55		62	3.19
Health product other than a medicine	27	21.4%	0.41		66	3.43
Alternative medical treatment	25	19.8%	0.47		64	3.38
New treatments	23	18.3%	0.57		60	3.17
Sports/ fitness related health	18	14.3%	0.34	(b)	72	3.47
Diagnosis	6	4.8%	0.46		58	3.00
Support group	6	4.8%	0.67		66	3.75
A second opinion	4	3.2%	0.44		64	3.25
Other	5	4.0%				

Total sample	126	218.3%	0.46		61	3.19

(a) Usefulness (mean score) Five point scale where; 1 = no use at all, 2 = somewhat useful, 3 = useful, 4 = very useful, 5 = extremely useful. Respondents were not restricted to whole numbers.

(b) Statistically significant difference (p < 0.05) based on non-overlap of the 95% confidence intervals calculated using t values and standard errors.

Responses add to more than 100% because some respondents gave more than one answer.

There were no statistically significant differences between income groups and genders for the mean values of search time and usefulness score. However, with respect to age there was a highly statistically significant difference for the mean search time (p < 0.001 analysis of variance F value 18.1). Search time increased with age: the over 65 years age group took 0.75 of an hour compared with 0.3 hours for the 16 to 25 years group. Usefulness scores were not statistically different between age groups. In only 22% of cases did a health professional (general medical practitioner, medical specialist, pharmacist, nurse, midwife, physiotherapist, osteopath, chiropractor, dentist or alternative health practitioner) suggest an internet website (general practitioner 9% and all others combined 13%).

The three most common actions taken as a result of the knowledge found were to talk with a family member, friend or workmate (58%), to contact a general medical practitioner (36%) and to change eating and/or drinking habits (33%). Thirteen percent did nothing as a result of the information found. If the topics "changed eating or drinking habits" "exercised more", "gave up smoking" and "relaxed more" are combined, 61% of the sample changed some aspect of their lifestyle. A total of 60% of respondents contacted a mainstream medical or health professional (general practitioner, medical specialist, osteopath, chiropractor, pharmacist, physiotherapist, nurse or midwife). When comparisons were made between different actions taken, no statistically significant differences were found with respect to search time or the value. There was however a statistically significant (p < 0.05) difference between scores for "did nothing" (2.59) and "bookmarked the website for future reference" (3.71) (table 5).

**Table 5:** Action taken by search time, value and usefulness

**Action**	**Number taking action**		**Search time**	**Value**	**Usefulness**	
	**N**	**%**	**Hours**	**$**	**Score**	(a)

Talked to family member, friend, neighbour, or workmate	73	57.9%	0.47	70	3.36	
Contacted general medical practitioner	45	35.7%	0.56	64	3.23	
Changed eating and/or drinking habits	42	33.3%	0.49	67	3.52	
Bookmarked the website for future reference	28	22.2%	0.44	74	3.71	(b)
Bought a health product from a health store	25	19.8%	0.36	63	3.32	
Exercised more	17	13.5%	0.43	75	3.18	
Bought a medicine or medical product from a pharmacist	17	13.5%	0.48	70	3.50	
Did nothing	16	12.7%	0.43	42	2.59	(b)
Contacted medical specialist	15	11.9%	0.53	71	3.13	
Gave up smoking	12	9.5%	0.46	65	3.50	
Contacted alternative health practitioner	7	5.6%	0.39	59	3.86	
Relaxed more	6	4.8%	0.54	71	2.83	
Contacted osteopath or chiropractor	5	4.0%	0.45	37	3.60	
Advice of health professional more likely to be followed	4	3.2%	0.69	91	3.50	
Contacted pharmacist	4	3.2%	0.44	55	3.63	
Contacted physiotherapist	4	3.2%	0.38	85	3.00	
Contacted support group	3	2.4%	0.50	82	3.83	
Bought a medicine or medical product over the internet	2	1.6%	0.48	115	4.00	
Contacted nurse/ midwife	2	1.6%	0.50	75	4.00	
Other	5	4.0%				
Total	126	263.5%	0.46	61	3.19	

(a) Usefulness (mean score) Five point scale where; 1 = no use at all, 2 = somewhat useful, 3 = useful, 4 = very useful, 5 = extremely useful. Respondents were not restricted to whole numbers.

(b) Statistically significant difference (p < 0.05) based on non-overlap of the 95% confidence intervals calculated using t values and standard errors.

Responses add to more than 100% because some respondents gave more than one answer.

A subset of 92 respondents provided the information set necessary to quantify the costs and benefits (table 6). The last time these respondents searched the internet for health information it took an average of 0.47 hours and the information found was assigned a mean usefulness score 3.2 (falling between useful and very useful). Respondents valued the information at an average of $60 (a proxy for the willingness to pay for the perceived benefit) which compares with the average general practitioner fee of $42 (inclusive of GST) [16]. The opportunity cost of the time taken to find the information on the internet was $12. The net benefit of the information found was $48 (benefit, $60 less cost $12 = net benefit $48). As the time cost of visiting a GP would be approximately one hour ($25) and the willingness to pay for the consultation ($42), the net benefit of a GP consultation would be $17 ($42 less $25).

**Table 6:** Net benefit of the information found

**Results from the survey**			
	N = 92		
	Mean	Standard deviation	95% confidence interval
Value score (# 1 to 5)	3.22	0.80	0.16
Benefit ($)	60 (a)	34.98	7.15
Search time (hours)	0.47	0.22	0.05
Annual income	47,000	24,432	4,992
Cost ($) (b)	12	8.48	1.73
Net benefit	48	33.98	6.94
			
**Sensitivity analysis**			

			Net benefits change from base case

Each of the following increased by 10% holding all else constant			
Benefit ($)			12%
Search time (hours)			-2%
Annual income			-2%

(a) Differs from tables 4 and 5 because the sample size vary between tables.

(b) Cost = annual income/47/40 × search time. Assumptions: 47 working weeks per year, 40 hours per working week, income group under $38,000 = $18,999.5, income group $38,000 to $60,000 = $49,000, and income group over $60,000 = $80,000.

Changing the valuation of benefits by 10% (holding all else constant) produced a 12% change in net benefits, while changing either of the cost components (search time and annual income) by 10% resulted in a 2% change in net benefits (table 6).

## Discussion

As almost all households have a telephone [17] random dialling should have produced a representative sample. However, the growth in telephone marketing and requests to support charities has increased citizen resistance to participation in a telephone survey which will have added some non-respondent bias. The survey was undertaken in Wellington and although the sample appeared to be over-represented by females and those people in the 26 to 45 years age group compared with Wellington data the differences did not indicate serious bias in the sample as they were not statistically significant (at the p = 0.05 level, Chi-square test). However, this does not prove that the sample is unbiased. We have no statistical data on those who refused to take part in the survey or those who had not searched the internet for health information.

The telephoned group had greater numbers than expected in the older age groups than the mall intercept respondents (Chi-square p < 0.05). Early evening (between 6 PM and 9 PM and Sunday afternoons were the best periods for the telephone survey, while the mall intercept interviews were conducted during the day. We consider the two data collection modes combined, captured a more representative sample than one mode alone. No other statistically significant differences between survey modes were found.

Although unavailability of data precluded an investigation of differences in income and educational status between the sample and the general population, international studies suggest that users of the internet have higher incomes and are younger than the general population. US and Australian researchers have found that those using the internet to find health information tended to be younger, were better educated, and had higher incomes than non-users [18-22].

The Wellington study sample is not necessarily representative of New Zealand as a whole. Wellington has higher income levels, lower unemployment, younger average age and higher levels of education than the rest of the country. Households in Wellington have greater access to the internet than other households in New Zealand [10]. The cost of a GP consultation in Wellington is probably higher than the New Zealand average but this was unable to be quantified as Wellington specific data were not available.

Most of our sample (83%) searched for information at home compared with 33% at work. A British survey [7] also found that most people (66%) searched the internet at home for health information and 28% from work. As personal use of the internet is discouraged in most work places and given the sensitive nature of some of the information sought it was not unexpected that most searches were conducted in the privacy of the home and that the information was for either themselves or a family member. The American Medical Association [23] note that personal privacy is the most important concern of users of medical information websites.

The top scoring search topic was general health and nutrition followed by information on a specific illness. Broadly similar results are reported in the international literature [7,24-27]. Very few health professionals had suggested a website to respondents.

Respondents valued the information highly (more than a general practitioner consultation), generally found it useful, and most took some action as a result of the knowledge gained. The three most frequent actions taken after finding the relevant information related to, talking with someone, changing some aspect of lifestyle or consulting a mainstream medical or health professional. A low proportion of searchers (13%) did nothing. A US study found that 59% of users of the internet for health information did not discuss the findings with their doctor, but those that did valued the information more highly than those that did not [19]. Australian research [22] found that 19% of searchers used the information as a second opinion, 16% discussed it with their doctor or pharmacist and 11% changed the way in which they managed their healthcare. Research in the UK [7] found that 93% of searchers found the information useful, 57% took some action to improve their health (mainly lifestyle changes), and 51% found information not provided by their doctor.

Our study is unique in that we asked Wellington respondents to assign a money value to the information found. The main limitations of our research are that respondents could have had difficulty in accurately recalling and estimating search times and values. Other limitations of the study were our inability to obtain precise income information (rather that income tax bands) and uncertainty that the sample was representative of the population that has used the internet to search for health information. We assumed that the value respondents assigned to information found could be used as a proxy for willingness to pay, and that the time costs of a search could be estimated from income levels. Sensitivity analysis revealed that net benefits of the information were sensitive to changes in the value of benefits but not to cost changes.

Although respondents stated that as a result of the information found they had made lifestyle or health related changes it is not known if these actions resulted in improved health outcomes and quality of life [22]. With the exception of clearly beneficial lifestyle changes such as reduced consumption of alcohol and tobacco, more information would be necessary to evaluate the health impact of the actions taken.

The internet is rapidly evolving, transcends national borders, and is not owned and cannot be controlled by any one country or individual. Dissemination of information via the internet is potentially lower cost than through traditional media. Content can be quickly updated and instantaneously published to the world-wide-web. However, the quality and unbiased nature of the information accessible on the world-wide-web cannot be guaranteed, and consumers may be unable to make an informed choice based on this information alone.

Poor quality information and advice on the world-wide-web, if followed, could be deleterious to health. Harm to individuals and wastage of health care resources may be caused through non compliance with health care professionals' advice, inappropriate/over/under treatment. Good quality information may however improve understanding of illness, increase compliance and reduce waste.

The internet has an important and growing role in the efficient and equitable provision of health-related information, and presents a partial solution to the problem of asymmetric information. Never-the-less governments should not necessarily devote scarce health care resources in attempting to regulate, censor or build their own health information websites that contain every conceivable item of health information. Governments can help consumers by providing a website containing information on treatment options for common illnesses, guidance to help citizens evaluate the quality of web-based information and links to other useful and reputable websites. The Harvard School of Public Health [28] has published a consumer's guide for evaluating health information and criteria for evaluating health related websites published in the BMJ [29] and the American Medical Association has developed guidelines for health information websites [23]. Such guidelines could be modified to suit the needs of New Zealand society and posted on a health information website. As a result of reviewing the literature and undertaking this study we have developed table 7. This table lists some important internet health information characteristics, consequences of variable information quality, and suggested criteria for an "official" health information website.

**Table 7:** Internet health information: characteristics, consequences of information quality and suggested criteria for an "official" health information website

**Characteristics of internet information**
• Rapidly evolving and uncontrolled growth of information
• Transcends international borders
• Is not owned and cannot be controlled by any individual, organisation or country
• Inability to restrict consumer access
• Quality of information is varied and cannot be guarantied
• High cost of constructing and maintaining a comprehensive and user friendly government health information website
• Potentially lower cost of information dissemination in comparison with traditional media
• Timeliness, in that information can be updated, inserted or deleted quickly
**Poor quality information may result in:**
• Non compliance with treatment recommendations of healthcare professionals that may incur additional costs of wasted healthcare resources and harm to consumers
• Inappropriate / over/ under treatment any of which could lead to additional cost and/ or harm
• Possible misinformation through lack of quality assurance
**Quality information should result in:**
• Improved understanding
• Improved compliance
• Reduced waste
**Criteria for a country wide "official" health information website:**
• Should provide:
• Up to date information on treatment options and prevention for common illnesses
• Links to useful and reputable websites irrespective of website owner
• Guidelines for evaluating information quality
• Contact for support groups
• Should not:
• Be all embracing and contain too much information
• Contain jargon and unfamiliar language
• Be biased towards a particular provider or funding agency

## Conclusion

Our research has shown that consumers value the internet as a health information source and use the information found to formulate future health and lifestyle strategies. An improved national health information website should be developed. However, more data on the information needs and Website preferences of stakeholders in the health care system (providers, funders and consumers) and impact on health outcomes are required to provide the information necessary to design such a website. Ideally this needs assessment should be publicly funded and not be conducted by any of the providers or funding agencies.

## Competing interests

The authors have no competing interests with the conduct and analysis of the research or with the publication of this article.

## Authors' contributions

WGS led the research project but all authors contributed to the design, analysis and interpretation of the data, drafting and revising the article and have given final approval for publication.

## Note on exchange rates

Mid rates end December 2004, Reserve Bank of New Zealand, NZ$1 = A$0.9315 and USA$0.7142
